# O-GlcNAcylation and Its Role in Cancer-Associated Inflammation

**DOI:** 10.3389/fimmu.2022.861559

**Published:** 2022-04-01

**Authors:** Muzi Ouyang, Changmeng Yu, Xiaolian Deng, Yingyi Zhang, Xudong Zhang, Fangfang Duan

**Affiliations:** ^1^Department of Pharmacology, School of Medicine, Sun Yat-sen University, Shenzhen, China; ^2^School of Anesthesiology, Xuzhou Medical University, Xuzhou, China

**Keywords:** O-GlcNAcylation, TME (tumor microenvironment), cancer inflammation, post-translational modification (PTM), HBP pathway

## Abstract

Cancer cells, as well as surrounding stromal and inflammatory cells, form an inflammatory tumor microenvironment (TME) to promote all stages of carcinogenesis. As an emerging post-translational modification (PTM) of serine and threonine residues of proteins, O-linked-N-Acetylglucosaminylation (O-GlcNAcylation) regulates diverse cancer-relevant processes, such as signal transduction, transcription, cell division, metabolism and cytoskeletal regulation. Recent studies suggest that O-GlcNAcylation regulates the development, maturation and functions of immune cells. However, the role of protein O-GlcNAcylation in cancer-associated inflammation has been less explored. This review summarizes the current understanding of the influence of protein O-GlcNAcylation on cancer-associated inflammation and the mechanisms whereby O-GlcNAc-mediated inflammation regulates tumor progression. This will provide a theoretical basis for further development of anti-cancer therapies.

## Introduction

The enzymatic process wherein carbohydrate moieties (referred to as glycans) get covalent attachment to proteins and lipids is defined as glycosylation. Protein glycosylation encompasses N-glycans, O-glycans, and proteoglycans. Until 1984, a novel form of protein O-glycosylation termed as O-linked β-N-Acetylglucosaminylation (O-GlcNAcylation) was firstly discovered by Hart and Torres (1). Unlike other types of protein glycosylation which glycosylate proteins either secreting from the cell, or residing in the extracellular plasma membrane, O-GlcNAcylation is an intracellular O-glycosylation, occurring in the nucleocytoplasmic and mitochondrial compartments. O-GlcNAcylation mediates the addition of a single monosaccharide, N-acetylglucosamine (GlcNAc), onto the hydroxyl groups of the amino acid serine or threonine residues of proteins to form a β-glycosidic bond. The donor substrate for protein O-GlcNAcylation is uridine diphosphate N-Acetylglucosamine (UDP-GlcNAc), which is the end product of the hexosamine biosynthetic pathway (HBP). As HBP integrates intermediate byproducts from lipids, amino acid and nucleotide metabolic pathways, O-GlcNAcylation is nutrition sensitive and is greatly impacted by the metabolism reprogramming in the tumor microenvironment.

To date, approximately 3000 human proteins have been confirmed to be O-GlcNAcylated (2). As one type of reversible protein post-translational modification, O-GlcNAcylation modulates protein functions mainly by regulating their enzyme activity, subcellular localization, protein stability, transcriptional activity and interaction with other proteins (3). Dysregulation in O-GlcNAc cycling has been implicated in the progression of various chronic human diseases including aging, obesity and diabetes, cardiovascular disease, neurodegenerative disorders, and carcinogenesis (4). Imbalanced levels of O-GlcNAcylation have been found in different kinds of cancers and contributing to various hallmarks of cancers such as tumor growth, metastasis, angiogenesis, cancer stem-like potential and metabolic reprogramming. Recent studies have identified O-GlcNAcylation is essential for the proliferation of corticotropic tumor cells (5). Increased O-GlcNAcylation together with mTOR pathways activation cooperate to increase Fatty acid synthase (FASN) expression to promote tumorigenesis of hepatic tumors (6). Moreover, O-GlcNAcylated MORC family CW-type zinc finger 2 (MORC2) at Thr556 is required for transcriptional activation of TGF-β1 to facilitate breast cancer cell migration and invasion (7). Inhibition of O-GlcNAcylation attenuates breast cancer stem-like cells (CSCs) potential (8). And genetic ablation of receptor for activated C kinase 1 (RACK1) O-GlcNAcylation at Ser122 dramatically suppressed angiogenesis in hepatocellular carcinoma (HCC) (9). Besides, β-catenin O-GlcNAcylation is accompanied by its nuclear translocation, thus enhancing the transcription of CEMIP to promote colorectal cancer metastasis *via* inducing glutamine metabolic reprogramming (10).

More than a century, accumulating evidence shows close association between chronic inflammation and increased risk of cancer progression and malignant development. Cancer-associated inflammation provides an immunosuppressive environment which aid the metabolic deregulations of the cancer cells, thus favoring tumor growth and metastasis (11). Moreover, metabolism reprogramming in tumor cells can also help sustain a chronic inflammation status (12). In comparation to normal cells, cancer cells have an altered cell metabolism to meet increased biosynthetic demand and energy requirements for tumor growth. Cancer cells took up high quantities of glucose and glutamine to sustain pools of various carbon intermediates. These metabolic changes accelerate the production of several molecules, such as lactate, reactive oxygen species (ROS) which simultaneously aid an inflammatory milieu (13). If the inflammation is unregulated and sustains to be chronic, various inflammatory cells will infiltrate and become active in the tumor microenvironment. These cells produce inflammatory mediators, such as growth factors, cytokines and chemokines and to stimulate tumor initiation and malignant growth.

Here, we focus on the latest research linking O-GlcNAcylation and cancer-associated inflammation and outline the underlying mechanisms of how O-GlcNAcylation drives cancer inflammation in the tumor microenvironment and promote cancer progression.

## O-GlcNAc: A Unique Type of Intracellular Monosaccharide Attachment

Before 1983, people never expected to find O-glycosylation occurring in the nucleocytoplasmic and mitochondrial compartments. O-GlcNAcylation was firstly reported in 1984 by Hart and Torres and they identified that the majority of O-glycosidically linked GlcNAc monosaccharide was inside the cells (1, 14). Their discovery was fascinating at that time. Primarily, O-GlcNAcylation was the first identified O-glycosylation which glycosylated proteins both in the nucleus and cytoplasm. Furthermore, similar to phosphorylation, O-GlcNAcylation was in a glycosylation and de-glycosylation dynamic equilibrium and was not as stable as proteoglycans in the extracellular matrix (15).

Additionally, there are several characteristics of this kind of post-translational modification. Initially, O-GlcNAcylation can only occur within the nucleocytoplasmic and mitochondrial compartments. Secondly, unlike “traditional glycosylation”, such as asparagine-linked or mucin-type O-glycosylation which can get extended into numerous different structures (16, 17), O-GlcNAcylation don’t get elongated to highly branched complicated structures (18). Instead, only one single GlcNAc was attached to the serine/threonine (S/T) residues on proteins by an O-linked β-glycosidic bond. Moreover, O-GlcNAcylation is a reversible and dynamic modification and mostly has a reciprocal relationship with phosphorylation. Numerous studies revealed O-GlcNAcylation occurs sequentially or reciprocally with phosphorylation on the same or nearby residues of numerous proteins. Here are some examples. For sequential crosstalk, Liu et al. determined that O-GlcNAcylation of Y-box binding protein 1(YB-1) at Thr126 was dependent on its phosphorylation at Ser102 to enhance YB-1 transcriptional activities in HepG2 cells (19). Tao and colleagues revealed that the TAK1 binding protein 3 (TAB3) was O-GlcNAcylated at Ser408 in triple negative breast cancer (TNBC), which was required for its Thr404 phosphorylation (20). In addition, Thr13 O-GlcNAcylation of MEK2 specifically enhanced its Thr394 phosphorylation together with downstream ERK1/2 activation to promote proliferation and migration of breast cancer cells (21). Recent study also identified Ser15 phosphorylation and Ser430 O-GlcNAcylation of PYGL were mutually reinforced under glucagon conditions in HCT116 cells (22). For reciprocal crosstalk, O-GlcNAcylation occupies the same site or adjacent positions to compete with phosphorylation and are capable to inhibit protein phosphorylation and vice-versa. Lei et al. determined O-GlcNAcylation of PFKFB3 could compete phosphorylation at the same Ser172 residue under hypoxic conditions in pancreatic cancer (23). Li et al. discovered Yes-associated protein (YAP) Ser109 O-GlcNAcylation promoted the malignant phenotypes in papillary thyroid cancer (PTC) cells by inducing YAP Ser127 dephosphorylation and activation (24). In NF-κB signaling, phosphorylation of p65 at Thr308 may prevent O-GlcNAcylation of p65 at Thr305 (25). Furthermore, O-GlcNAcylation/Phosphorylation crosstalk can even occur whereby the two PTMs are situated at quite distal sites, such as O-GlcNAcylation of endothelial nitric oxide synthase (eNOS) at Ser615 could interfere its phosphorylation at Ser1177 (26). LATS2 O-GlcNAcylation at Thr168 and Thr436 inhibited its phosphorylation at Ser872 and Thr1041, which has a decisive effect on LATS2 activation (27). Considering all of the above mentioned, this kind of O-GlcNAcylation/Phosphorylation relationship was termed “yin-yang model” (28). Moreover, apart from phosphorylation, O-GlcNAcylation has interplay with other posttranslational modifications, such as ubiquitination, acetylation and methylation (29–31).

Last but not least, another characteristic of O-GlcNAcylation is that in contrast to phosphorylation where there exist numerous kinases or phosphatases mediating to add or remove phosphorylation, the addition and removal of O-GlcNAcylation to target proteins are regulated by one single O-linked-β-N-acetylglucosamine transferase (O-GlcNAc transferase, OGT) and one single β-N-Acetylglucosaminidase (O-GlcNAcase, OGA), respectively. OGT uses UDP-GlcNAc to catalyze O-GlcNAc addition while OGA modulates O-GlcNAc removal from target proteins. Both OGT and OGA are highly conserved across evolution (32, 33). In mice, deletion of OGT is lethal at the embryonic level and conditional disruption of the OGA gene causes perinatal lethality, indicating the key role of O-GlcNAcylation in regulating fundamental cellular biological processes (34, 35).

## O-GlcNAcylation: A Metabolic Link Between Nutrition Homeostasis and Cancer-Associated Inflammation

One hallmark of cancer progression is altered metabolic state, shifting from oxidative phosphorylation to aerobic glycolysis which is termed as Warburg effect, characterized by large scale of glucose uptake (36). This phenomenon not only promote rapid ATP synthesis, but also help sustained production of glycolytic carbon intermediates required for the increased biosynthetic demands needed by the rapidly dividing cancer cells (36). The abundance of glucose in cancer cells primarily enters glycolysis, and also increases flux to glucose branched pathways, such as the hexosamine biosynthetic pathway (HBP).

**Figure 1:**
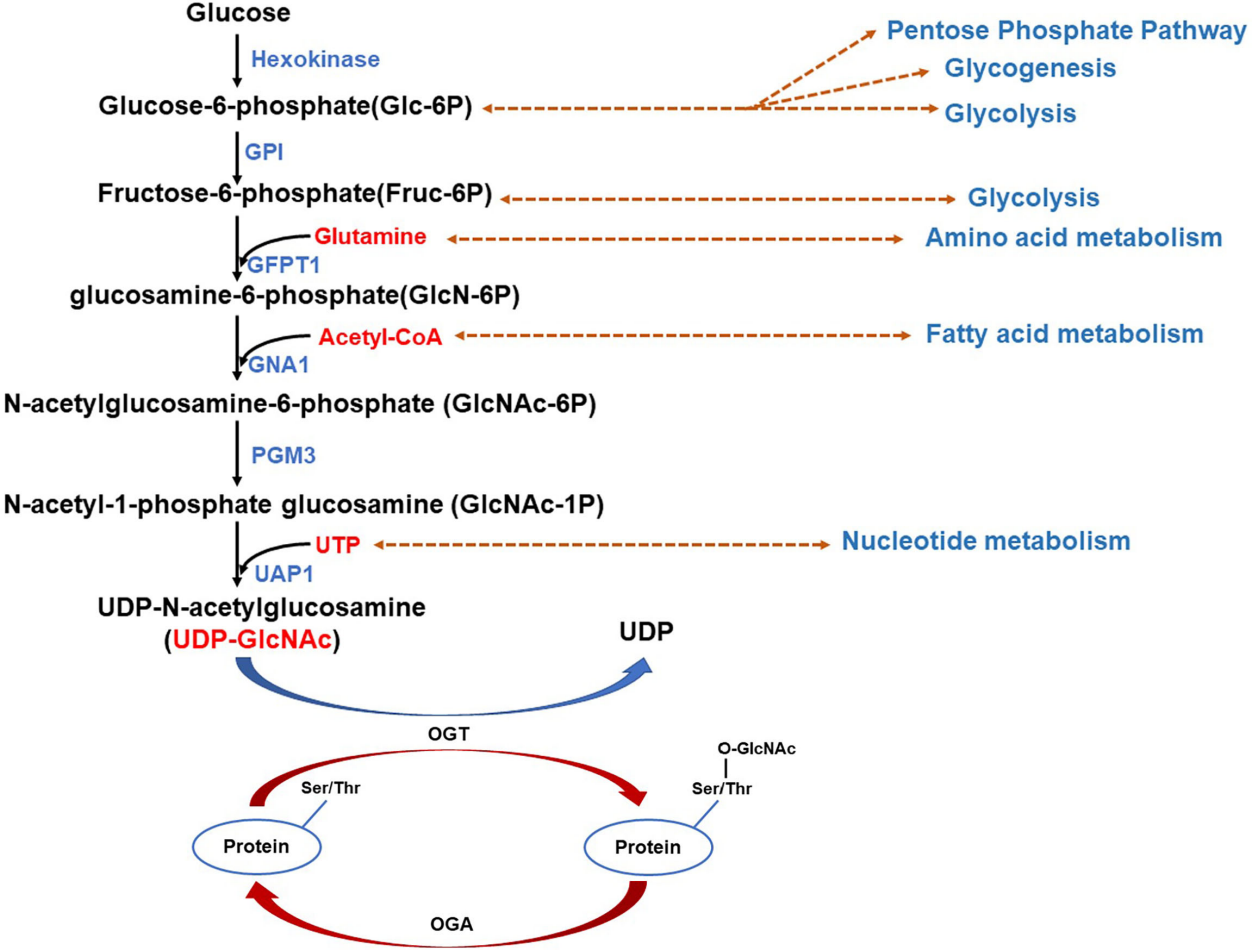
Schematic illustration of the hexosamine biosynthesis pathway (HBP) integrating different metabolic intermediates fluxing to UDP-N-Acetlyglucosamine (UDP-GlcNAc). Glucose is phosphorylated by hexokinase yielding glucose-6-phosphate (Glc-6P) which can be converted to fructose-6-phosphate (Fruc-6P) by glucose-6-phosphate isomerase (GPI). Both Glc-6P and Fruc-6P can be metabolized by glycolysis or the HBP. The rate-limiting enzyme of HBP: Glutamine-fructose 6-phosphate aminotransferase (GFPT1 or GFAT) mediates the diverge to HBP, conversing Fruc-6P to glucosamine-6-phosphate (GlcN-6P) from glutamine, the amide donor. Additionally, GFAT mRNA and protein expression was upregulated by saturated fatty acids (palmitate and stearate), whereas inhibition occurs owing to feedback from its enzymatic product GlcN-6P. Next, acetyl-CoA (AcCoA) and GlcN-6P are converted by D-glucosamine-6-phosphate N-Acetyltransferase (GNA1) to CoA and N-Acetylglucosamine-6-phosphate (GlcNAc-6P). After that, another enzyme, GlcNAc phosphomutase (PGM3), converts GlcNAc-6P to N-Acetyl-1-phosphate glucosamine (GlcNAc-1P), employing glucose-1,6-bisphosphate as a co-factor. The nucleoside (UTP) is then added to the sugar by UDP-N-Acetylhexosamine pyrophosphorylase (UAP1) yielding UDP-N-Acetlyglucosamine(UDP-GlcNAc). Glucose-6-phosphate isomerase (GPI); Glutamine-fructose 6-phosphate aminotransferase (GFPT1 or GFAT); D-glucosamine-6-phosphate N-Acetyltransferase (GNA1); GlcNAc phosphomutase (PGM3); Nucleoside (UTP); UDP-N-Acetylhexosamine pyrophosphorylase (UAP1).

HBP integrates various metabolic inputs, such as glucose, amino acid, fatty acid, nucleotide, to promote the synthesis of UDP-GlcNAc (37) (**Figure 1**). UDP-N-Acetlyglucosamine (UDP-GlcNAc) is the end product of HBP and is also the substrate for O-GlcNAcylation. As HBP integrates different metabolic intermediates fluxing to UDP-GlcNAc, O-GlcNAcylation act as a “nutritional sensor” (38, 39). Therefore, O-GlcNAcylation has been considered to serve in the regulation of cellular signaling, transcription in the cancer cells, as well as the adjacent stromal and inflammatory cells in response to nutrients and stresses in the TME. The signaling pathways activated in these cells in TME convert environmental cues into intracellular events, such as immune cell activation and inflammation to form an immunosuppressive inflammatory TME to promote cancer progression.

The relationship between inflammation and cancer was firstly discovered in 1893 by Rudolf Virchow who found leucocytes in neoplastic tissues and make it clear that inflammation accompanies with cancer (40). Cancer-associated inflammation can fall into two categories: cancer extrinsic inflammation and cancer intrinsic inflammation. Cancer extrinsic inflammation is driven by chronic long-term inflammatory conditions which predispose to cancer (41). There are some risk factors accounting for the cancer extrinsic inflammation, composing of infections from bacterial and viral, obesity, autoimmune diseases, tobacco smoking, and excessive alcohol consumption. For example, infection with Helicobacter pylori positively correlates with tumorigenesis of gastric cancer and gastric mucosal lymphoma. Some autoimmune diseases, such as Crohn’s disease (CD) is associated with colon cancer progression. Around 15%–20% of all cancer cases show this kind of precancerous inflammatory conditions present before a malignant change occurs (41). All of these inflammatory conditions form a constant inflammatory state, causing precancerous inflammation lesions which will precede the development of cancer malignancy.

However, most cancers are not developed from long-lasting chronic inflammation (42). In contrast, cancer cells recruit immune cells and secrete inflammatory mediators to reshape the tumor microenvironment (TME) to promote cancer intrinsic inflammation (41). Cancer intrinsic inflammation or cancer-elicited inflammation is defined as inflammation driven by genetic events that cause neoplasia (39). It is elicited by genetic and/or epigenetic mutation (Oncogenes) that drives a tumor-promoting inflammatory milieu which involves the recruitment and activation of inflammatory cells. Cancer cells, as well as the adjacent stromal and inflammatory cells participate in well-orchestrated reciprocal interactions to form an inflammatory TME to promote cancer progression.

**Figure 2:**
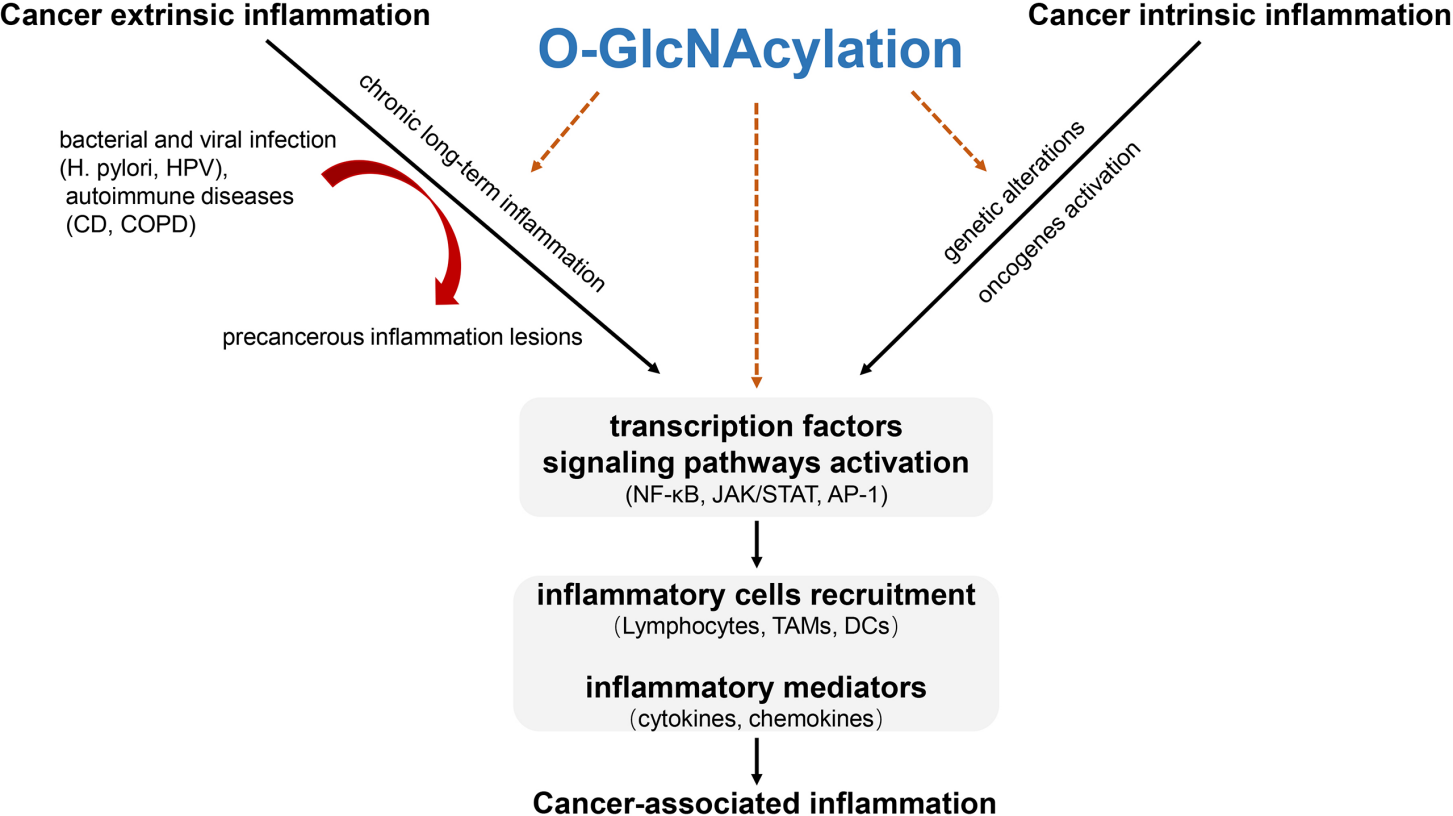
The role of O-GlcNAcylation involved in cancer-associated inflammation. Cancer-associated inflammation can fall into cancer extrinsic inflammation and cancer intrinsic inflammation. Cancer extrinsic inflammation is driven by long-lasting chronic inflammation to form precancerous inflammation lesions while cancer intrinsic inflammation is elicited by genetic and/or epigenetic mutation to form an inflammatory milieu. The two ways converge to promote transcription factors-mediated signaling pathways activation, thus facilitating inflammatory cells recruitment and inflammatory mediators’ production. O-GlcNAcylation promotes precancerous inflammation and acts as a key orchestrator at the intersection of the intrinsic and extrinsic inflammation through O-GlcNAcylating key transcription factors and functional proteins in inflammatory cells activation to trigger cancer-associated inflammation in tumor microenvironment. H. pylori, Helicobacter pylori; HPV, Human papillomavirus; CD, Crohn’s disease; COPD, Chronic obstructive pulmonary disease; TAMs, Tumor associated macrophages; DCs, Dendritic cells.

Both cancer extrinsic and intrinsic inflammation results in transcription factors and core signaling pathways activation to modulate the inflammatory response through soluble mediators (cytokines, chemokines) and cellular components (e.g. tumor-associated macrophages). During these processes, O-GlcNAcylation acts as a key orchestrator at the intersection of the intrinsic and extrinsic pathways to trigger immunosuppression in tumor microenvironment, thus favoring tumor tumorigenesis (**Figure 2**).

## O-GlcNAcylation Implicates in Precancerous Inflammation Lesions of Different Cancer Types

Epidemiological studies have attributed up to 25% of all cancers are related to chronic infection, termed as cancer extrinsic inflammation (43–45). Numerous studies identified that chronic inflammation is an vital risk factor for the development of cancer progression, such as Gastric cancer [Helicobacter pylori (H. pylori)] (46), Colorectal cancer [Inflammatory bowel disease (47)], Hepatocellular carcinoma [Hepatitis B/C virus (48)], Prostate cancer [or Prostatitis (49)], Pancreatic cancer [Pancreatitis (50)], Cervical cancer (Human papillomavirus (HPV) (51), Lung cancer [(Chronic obstructive pulmonary disease(COPD)] (52), Bladder cancer (Schistosoma haematobium) (53). All of these inflammatory conditions form a constant inflammatory state, causing precancerous inflammation lesions which will precede the development of cancer malignancy.

It has been shown that H. pylori infection creates an inflammatory environment, providing a favorable role in gastric cancer oncogenesis and is well known to be associated with the development of precancerous lesions such as chronic atrophic gastritis (AG) (54). Tae and colleagues determined both O-GlcNAcylation and OGT expression in the epithelium and mononuclear inflammatory cells from biopsied tissues of chronic gastritis (55). They also demonstrated that elevated expression of OGT and O-GlcNAcylation are found in H. pylori-infected chronic gastritis than those in chronic gastritis without H. pylori infection. Their findings demonstrated the importance of O-GlcNAcylation in the development of gastric cancer precancerous lesions.

Inflammation is also an established risk factor for colorectal cancer (CRC) (56). Ulcerative colitis (UC) and Crohn’s disease (CD) are common types of inflammatory bowel disease (IBD) which causes inflammation and irritation of the gastrointestinal tract, thus increasing risk for bowl cancer (57). Yang and colleagues discovered elevation of O-GlcNAcylation was found in dextran sulfate sodium (DSS)-induced colitis and increased O-GlcNAcylation promotes the progression of colitis-associated colorectal cancer (58). Elevated O-GlcNAc levels also showed in intestinal epithelial tissues of active CD patients and adherent-invasive Escherichia coli (AIEC) LF82-infected mice (59). Inhibition of O-GlcNAc protects mice from DSS (dextran sulfate sodium)- and AIEC LF82-induced intestinal inflammation. Moreover, gut microbiota also influences intestinal inflammatory physiology. The enzyme OGAs from gut microbiota help hydrolyse O-GlcNAcylated proteins in host cells, thus suppressing inflammatory response in the gut and protecting mice from chemically induced colonic inflammation (60). These results demonstrated O-GlcNAcylation provides a favorable role in IBD-induced chronic intestinal inflammation and inhibition of O-GlcNAc from gut microbiota can reverse colonic inflammation.

Chronic hepatitis B virus (HBV) infection contributes to at least 50% cases of Hepatocellular carcinoma (HCC) worldwide (61). HBV infection stimulates the host immune response and drives liver chronic necro-inflammation to promote hepatocarcinogenesis (62). Hu et al. revealed total O-GlcNAcylation levels got markedly higher regulation in liver tissues from patients with chronic hepatitis B (CHB) than in those from normal controls (63). HBV infection upregulated glucose transporter 1 (GLUT1) expression which facilitated glucose uptake, thus leading to an increase in protein O-GlcNAcylation. Pharmacological or transcriptional inhibition of HBP and O-GlcNAcylation enhanced HBV replication because O-GlcNAcylated SAMHD1 at Ser93 stabilizes SAMHD1 and enhances its antiviral activity. The findings reveal a link between HBP, O-GlcNAc modification, and host antiviral immune response against HBV.

Inflammation have been reported to promote the progression of benign prostatic hyperplasia (BPH) (64). Meta-Analysis suggested BPH is associated with an increased risk of prostate cancer, especially in Asian BPH patients (65). Gu et al. identified that elevated O-GlcNAcylation levels induces malignant transformation of nontumorigenic benign prostatic hyperplasia (BPH) cells, enhancing migratory and invasive ability through inhibiting the formation of the E-cadherin/catenin/cytoskeleton complex (66). O-GlcNAcylation is shown to be an inducer of prostate cancer progression.

Patients with acute pancreatitis (AP) characterized by inflammation had an increased risk of pancreatic cancer (67). Zhang et al. found that both OGT and O-GlcNAcylation were upregulated in acute pancreatitis cell model which was constructed with caerulein-stimulated AR42 J rat pancreatic acinar cells. Reducing the expression of OGT attenuated the severity of inflammation while O-GlcNAc upregulation increased the AP severity (68). In this AP model, both NF-κB subunit p65 and its upstream activating kinases IKKα were O-GlcNAcylated, which are responsible for the inflammatory NF-κB activation during acute pancreatitis. The results demonstrate that OGT-mediated O-GlcNAcylation promotes NF-κB-mediated inflammation in pancreatic acinar cells to promote the progression of AP.

Persistent genital high-risk human papillomavirus (HPV) infection accounts for about 99.7% of cervical cancer (69). Histological analysis revealed a higher degree of inflammation in biopsies of cervical mucosa from high risk (HR)−HPV−infected females, accompanied with an increased infiltration of neutrophils and lymphocytes into the epithelium, comparing with those who were uninfected (70). Cervical cancer can origin from persistent HPV lesions through the action of two HR HPV genes, *E6* and *E7* while HPV *E6/E7* gene transcription can be upregulated by OGT though O-GlcNAc modification of HCF-1 in cervical cancer cells (71). Furthermore, levels of O-GlcNAc and OGT in HPV-associated cervical neoplasms were markedly increased relative to the normal cervix. Pharmacological inhibition of O-GlcNAcylation impaired HPV-mediated cervical carcinoma cells viability and transformation (72). Thus, O-GlcNAcylation serves as an essential regulator to facilitate HPV-associated malignancies.

Chronic obstructive pulmonary disease (COPD) is a characterized by persistent respiratory symptoms and enhanced inflammatory response (73). People with COPD have higher risk (4- to 6-fold) of developing lung cancer, ignore of the patients’ history, such as smoking history, age and sex (74, 75). HBP/O-GlcNAc modification activation is stimulated in human bronchial epithelial cells by FGF23 through the PLCγ signaling pathway, leading to NFAT activation and increased secretion of IL-6 which contribute to the progression of pathogenesis of chronic inflammatory airway diseases such as COPD (76). These findings revealed the link whereby FGF23 and the augmentation of O-GlcNAc levels regulate chronic airway inflammation.

Taken together, O-GlcNAcylation serve as a key regulator to facilitate the progression of these precancerous inflammation lesions, thus providing a tumor-supporting chronic immunosuppressive microenvironment for tumor initiation, growth and progression.

## O-GlcNAcylation and Inflammation- Related Signaling Pathways

During both cancer intrinsic and extrinsic inflammation, a wide array of intracellular signaling transduction pathways are often dysregulated to stimulate malignant transformation. Transcription factors (TFs) (Nuclear factor-κB (NF-κB), STAT1/STAT3, HIFs, AP-1, and Nrf2) orchestrators various inflammation-related signaling pathways to modulate the inflammatory response, through inflammatory mediators (such as cytokines, chemokines) and immune cells infiltration (tumor-associated macrophages), thus promoting tumorigenesis. The following sections illustrate the role of O-GLCNAC in linking TFs with cancer-associated inflammation.

NF-κB activation plays central roles in inflammatory and immune responses (77). Nuclear factor-κB (NF-κB) consists of five master transcription factors, including p65 (RelA), RelB, c-Rel (Rel), p50/p105 (NF-κB1), and p52/p100 (NF-κB2) in mammals (78). In most cell types, NFκB is composed of p65 and p50 and is localized in the cytosol where it binds inhibitor (IκB). NF-κB can be activated by various stimuli which lead to the activation of the inhibitor of κB (IκB) kinase (IKK) complex. The activated IKK complex mediates phosphorylation of IκB for proteasomal degradation (79). Thus, the free NF-κB translocate from the cytoplasm to the nucleus, bind to DNA elements and activate the expression of target genes.

Studies revealed site specific O-GlcNAcylation is implicated in NF-κB pathway mediated inflammation. O-GlcNAcylation of NF-κB p65 on Thr352 decreases its binding to IκBα is required for transcriptional activity under hyperglycemic conditions (80). Yang et al. determined pancreatic ductal adenocarcinoma (PDAC) is supported by oncogenic NF-κB transcriptional activity and mutation of two p65 O-GlcNAc sites (T322A and T352A) attenuated the growth of PDAC (81). Apart from tumor growth, O-GlcNAcylation of NF-κB subunit p65 also promotes lung metastasis of cervical cancer cells *via* upregulating CXCR4 expression (82). Point mutated p65 at Thr322 or Thr352 in HeLa cells decreased CXCR4 expression compared to transfection with wild-type p65, indicating site specific p65 O-GlcNAcylation contributes to the regulation of CXCR4 expression in cervical cancer cells (82). Furthermore, O-GlcNAcylation upregulates matrix-metalloproteinases (MMPs) expression to enhance cholangiocarcinoma (CCA) cell migration and invasion *via* inducing NF-κB p65 nuclear translocation (83). However, whether up-regulation of MMPs mediated by O-GlcNAcylation in CCA is dependent on p65 O-GlcNAcylation at Thr322 and Thr352 is not investigated in this paper (83). In addition, there is some discrepancy between results from two studies on p65 O-GlcNAcylation in colon cancer progression. Yang et al. discovered p65 O-GlcNAcylation at Thr322 and Thr352 is required for transcriptional activation of p65 target genes (*IL-6*, *TNF-α* and *MCP1*) and promotes colonic inflammation and tumorigenesis (58). Nevertheless, Hirata and colleagues identified O-GlcNAcylation could protect from inflammation-induced colon carcinogenesis *via* suppressing NF-κB signaling (84). Their inconsistent findings could be from the difference of mouse model they used. Yang et al. implemented an Oga+/– mouse model while Hirata et al. utilized OGT-transgenic (OGT-Tg) mice. Besides subunit p65, O-GlcNAcylation of NF-κB subunit c-Rel can also get O-GlcNAcylated. O-GlcNAcylation of NF-κB subunit c-Rel at Ser350 was required for the DNA binding and transactivation. Blocking O-GlcNAcylation of this residue abrogated c-Rel-mediated expression of the cytokine-encoding genes *IL2, IFNG*, and *CSF2* to promote T cell-mediated autoimmunity (85).

Moreover, the activation of NF-κB and IKK requires an upstream kinase complex consisting of TGFβ (transforming growth factor-beta)-activated protein kinase (TAK1) and adaptor proteins such as TAK1 binding protein 1 (TAB1), TAB2, TAB3 (86). Ser395 O-GlcNAcylation of TAB1 is essential for full TAK1 activation to induce NF-κB activation, thus favoring IL-6 and TNF-α production (87). Tao et al. revealed O-GlcNAcylation of TAB3 at Ser408 promotes triple negative breast cancer cell migration, invasion and is correlated with patient poor prognosis (20). The IKK complex is composed of two catalytic subunits, IKKα and IKKβ. O-GlcNAcylation of IKKβ sustained the TNFα-dependent IKKβ activation, thus stimulating NF-κB signaling and increased IKKβ expression is essential for cell viability in prostate cancer (88, 89). These studies collectively provide insights into the mechanism of O-GlcNAcylation regulating upstream signal transductors in NF-kB activation and suggests its important implications in tumor growth and metastasis.

Along with NF-κB, the Janus kinase/signal transduction and activator of transcription (JAK-STAT) signaling pathway is implicated in regulating cytokine-dependent inflammation and immunity in carcinogenesis (90). The binding of various ligands, usually cytokines, to cell-surface receptors cause the receptor to activate JAKs, which phosphorylate tyrosine residues on the receptor thereafter and recruit STATs proteins. Tyrosine-phosphorylated STATs dimerize and are then transported into the nucleus to transactivate target genes. There are four JAK proteins: JAK1, JAK2, JAK3, and TYK2. The STAT family comprises seven members: STAT1, STAT2, STAT3, STAT4, STAT5A, STAT5B, and STAT6 (91). STAT3 acts as a key mediator of intestinal inflammation and tumorigenesis (92). Studies determined O-GlcNAcylation of STAT3 at Thr717 negatively regulates its phosphorylation in colon macrophages, which promote colonic inflammation and inflammation-driven tumorigenesis (92). O-GlcNAcylation of STAT5A at Thr92 accompanied enhanced STAT5 tyrosine phosphorylation, driving oncogenic transcription to induce myeloid transformation (93). Furthermore, STAT5 can directly regulate hypoxia inducible factor (HIF) 1β, whereas STAT3 directly controls HIF1α (94). HIF1α was reported to augment inflammation in a mice model of proximal colon cancer (95). Elevated O-GlcNAcylation stabilized HIF-1α levels, protecting breast cancer cells from ER stress-mediated apoptosis (96). These studies indicated O-GlcNAc-modified STATs proteins can regulate cancer-related inflammation independently or through modulating HIFs proteins production.

The activator protein-1 (AP-1) family of transcription factors consist of multiple Jun (c-Jun, JunB, and JunD) and Fos (c-Fos, FosB, Fra1, and Fra2) members (97). Similar to NF-κB, AP-1 can also bind promoters of inflammatory mediators (IL6, IL8) to promote cancer-associated inflammation (98). Studies suggest that OGT plays an oncogenic role in non-alcoholic fatty liver disease-associated hepatocellular carcinoma (NAFLD-HCC) through activating JNK/c-Jun/AP-1 cascade by increasing p-JNK, p-c-Jun protein expression and AP-1 activation. In keeping with this, NF-κB cascade was activated and NF-κB DNA binding activity was enhanced to induce downstream genes (TNF-α) expression (99). As JNK and NF-κB signaling pathways are the major endoplasmic reticulum (ER) stress-related oncogenic signaling pathways, OGT acts as a mediator to induce ER stress to promote NAFLD-HCC. Mechanistically, OGT regulates lipid metabolism through increasing palmitic acid production and reactive oxygen species (ROS), thereby activating ER stress, and ER stress-related JNK/c-Jun/AP1 and NF-κB pathways. OGT may serve as a potential therapeutic target in NAFLD-HCC.

Taking together, these findings provide compelling evidence for cooperative relationship between O-GlcNAcylation and NF-κB, STATs, HIF-1, AP-1-mediated cancer-related signaling pathway.

## O-GlcNAcylation and Tumor Stromal Cells in Tumor Microenvironment (TME)

**Figure 3:**
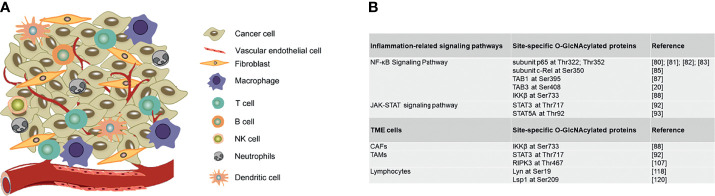
Site-specific O-GlcNAcylation of functional proteins in regulating inflammation-related signaling pathways and stromal cells in TME. **(A)** Graphic representation of TME cells. **(B)** Site-specific O-GlcNAcylated proteins in regulating inflammation-related signaling pathways and stromal cells in TME. TME, Tumor microenvironment; CAFs, Cancer-associated fibroblast; TAMs, Tumor associated macrophages.

A tumor contains not only a group of cancer cells, but also a heterogeneous collection of infiltrating and resident host cells, such as stromal fibroblasts, endothelial cells and immune cells like macrophages and lymphocytes and the non-cellular extracellular components such as cytokines, growth factors, collagen, fibronectin, laminin, among others (100, 101). A dynamic and reciprocal relationship develops between cancer cells and resident host cells of the tumor microenvironment to create an immunosuppressive environment to promote tumor growth and metastasis. Here we illustrate how O-GlcNAcylation regulates TME cells functions in tumor immune microenvironment and also summarize site-specific O-GlcNAcylation of functional proteins in regulating inflammation-related signaling pathways and stromal cells in TME (**Figure 3**).

### Vascular Endothelial Cells

To maintain vessel homeostasis, tumor endothelial cells (TECs) not only activate on environmental stressors, such as pro-angiogenic signals and hypoxia to initiate tumor angiogenesis, but also regulate peripheral immune cell trafficking into the tumor compartment (102). Hypoxia is a common physiological and pathophysiological occurrence in tumors and coexist with inflammation. Studies revealed hypoxia regulates vascular inflammatory response through upregulation of 26S proteasome activity and downregulate OGT expression in vascular endothelial cells (103). Mechanically, hypoxia generates reactive oxygen or nitrogen species (ROS/RNS) to enhanced 26S proteasome functionality, favoring E3 ubiquitin ligase β-TrCP1-mediated OGT degradation to facilitate vascular endothelial inflammation. Up-regulation of OGT can prevent this hypoxia-mediated inflammation in vascular endothelial cells, suggesting OGT may be targeted to treat diseases characterized by hypoxic inflammation (103).

### Fibroblasts

Inflammation is often accompanied by the recruitment of fibroblasts and the induction of fibrosis. Cancer-associated fibroblast (CAFs) promote the deposition of collagen and various ECM components in the TME to facilitate tumorigenesis (104). Kawauchi and colleagues revealed that O-GlcNAcylation of IKKβ occurred in both p53-deficient mouse embryonic fibroblasts (MEFs) and transformed human fibroblasts (88). O-GlcNAcylation of IKKβ occurred at Ser733 sustained the TNFα-dependent IKKβ activity and enhance NF-κB activity. Thus, these results implicate O-GlcNAcylation of IKKβ as a central component linking glucose metabolism to IKK–NF-κB signaling pathway, providing a favorable role in inflammation-associated tumor development.

### Macrophages

Tumor associated macrophages (TAMs) are derived from circulating monocytes and are divided into two subtypes: M1 and M2 macrophages. M1 macrophages are pro-inflammatory and can produce pro-inflammatory cytokines such as interleukin-1β (IL-1β), IL-6, IL-12, IL-23, and TNF-α while M2 macrophages are anti-inflammatory and produce anti-inflammatory cytokines such as IL-10 and TGF-β (105). O-GlcNAcylation promotes opposing effects in TAMs. In colorectal cancer, increased O-GlcNAcylation skewed macrophage polarization to a M2-like phenotype to enhance cancer progression and immune evasion (106). O-GlcNAcylation of STAT3 at Thr717 regulates its phosphorylation in colon macrophages negatively, which was accompanied by exacerbated colonic inflammation and inflammation-driven carcinogenesis (92). Nevertheless, a decreased HBP activity and protein O-GlcNAcylation was observed in LPS-stimulated macrophages where O-GlcNAcylation of RIPK3 at Thr467 suppresses RHIM-mediated RIPK3-RIPK1 interaction and downstream RIPK3 kinase activation, thus inhibiting inflammatory responses and inflammation-associated necroptosis (107). Moreover, LPS treatment increases the interaction of OGT with transcriptional corepressor mammalian Sin3A (mSin3A) to inhibit inducible nitric oxide synthase (iNOS) transcription while GlcN-induced hyper-O-GlcNAcylation inhibits LPS-driven activation of NF-κB and iNOS expression in RAW264.7 cells, which are monocyte/macrophage-like cells derived from BALB/c mice. These results indicate that OGT functions as a transcriptional repressor and exerts inhibitory effects on LPS-induced inflammation (108). Taken together, the above results collectively show O-GlcNAcylation appears to have opposite functions in inflammatory responses in macrophages. On some occasions, O-GlcNAcylation promotes inflammatory responses in macrophages, and in certain scenarios may has an anti-inflammatory function.

### Lymphocytes

Tumors was infiltrated with diverse immune cells and there are three main types of lymphocytes: T cells, B cells, and Natural killer (NK) cells. Within the tumor microenvironment there are several distinct populations of T cells that influence tumorigenesis, comprising of cytotoxic (CD8+) T cells and helper (CD4+) T cells which could differentiate into Th1, Th2, Th17, and regulatory T cells (Tregs) Upon activation (109).

Following tumor infiltration, naive CD8+ T cells are differentiated into effector CD8+ T cells and further differentiated and activated into cytotoxic and memory CD8+ T cells (110). It has been reported that O-GlcNAcylated proteins enriched in Murine effector and memory-like CD8+ T cells. O-GlcNAcylation in effector CD8+ T cells promote transcription and translation essential for the regulation of fast cell proliferation, while protein O-GlcNAcylation in memory-like CD8+ T cells is involved in the transcription, mRNA processing, and translation during memory T cell formation (111). The main cluster of proteins identified O-GlcNAcylation between effector- and memory-like T cells consisted primarily of histones, identifying O-GlcNAc critical role as part of the “histone code” in T cell subgroups. Exosomes (30–100 nm small lipid bilayer extracellular vesicles) are secreted by various kinds of cells and play an important role in intercellular communication due to their ability to transfer proteins, lipids and nucleic acids to surrounding recipient cells. Numerous studies have demonstrated cancer-derived exosomes alter immune players in the TME to induce a pro-tumoral environment to facilitate tumor progression. Yuan et al. identified OGT in exosome from Esophageal carcinoma stem cells (ECSCs) promoted the expression of PD-1 in neighboring CD8+ T cells, thus favoring ECSCs the ability of immune escape (112). Moreover, O-GlcNAcylation also play an important role in the differentiation of Th cells. Elevated O-GlcNAcylation promotes increased IL-17A production in CD4+ T cells polarized to the Th17 lineage (113). Administration of Thiamet-G, which is an O-GlcNAcase inhibitor, increased the binding of transcription factor RORγt for commitment of the Th17 lineage, to *IL-17* promoter and therefore promotes IL-17 production and Th17 differentiation (114). Subsequently, pro-inflammatory responses were enhanced by Th17 cells. In Treg cells, O-GlcNAcylation stabilizes FOXP3 and activates IL-2/STAT5 signaling, thus supporting Treg cells lineage stability and promoting the suppressive program of Treg cells (115). Collectively, O-GlcNAcylation is therefore shown to be an essential regulator in T cell development, transformation and differentiation. In addition, Loss of OGT blocked T cell progenitor renewal, malignant transformation and peripheral T cell clonal expansion (116).

B cells contribute to adaptive immune responses responsible for antibody production and promoting T cell activation *via* antigen presentation. Compared to T cells, relatively few B cells infiltrated in the tumor microenvironment. B cells are typically found in lymph nodes in close proximity to the TME (100). Following antigen engagement with B-cell receptors (BCRs), a series of signaling cascades involving the activation of spleen tyrosine kinase (Syk) and Lck/Yes-related Novel protein tyrosine kinase (Lyn) are triggered, which further activate downstream pathways, such as PLC-γ2/calcium/NFAT, IKK/NF-κB to induce transcription of genes that regulate the functions of activated B cells (117). Studies determined that O-GlcNAcylation of Lyn at Ser19 is detrimental for Lyn activation and Syk interaction in BCR-mediated B-cell activation (118). NF-κB and NFAT can be O-GlcNAcylated by OGT which mediates their translocation to the nucleus, sensitizing T and B lymphocytes toward activation (119). Lack of OGT in B-cell development not only inhibits activation of BCR signaling, but also enhances apoptosis of mature B cells, thus perturbing B-cell homeostasis. In addition, apoptosis initiates following BCR activation to eliminate auto-reactive B cells. O-GlcNAcylation of a pro-apoptotic regulator Lsp1 at Ser209 is required for the recruitment of its kinase, PKC-β1, to stimulate Ser243 phosphorylation, leading ERK activation and downregulation of BCL-2 and BCL-xL which initiate B-cell activation and apoptosis (120).

Natural killer (NK) cells are a population of innate lymphoid cells involved in immunosurveillance of solid tumors. These cells possess powerful cytotoxic activity orchestrated by an intricate network of signals to control tumor growth and mediate anti-metastatic effect in tumors (121). In TME, high density of tumor-infiltrating NK cells predicts favorable prognosis in multiple human tumors (122, 123). Soluble HLA-G1α chain (sHLA-G1α chain) are secreted proteins to exhibit systemic immunoinhibitory functions in peripheral blood. It has been shown that O-GlcNAcylation decreased during NK cell cytotoxicity and that GST-sHLA-G1α chain could inhibit the decrease of O-GlcNAc level during the process to inhibit NK cell cytotoxicity, thus inducing immunotolerance (124). These findings suggest O-GlcNAcylation important implications in the cytotoxic signal transduction of NK cells. Upon activation, NK cells release cytotoxic granules containing perforin, cathepsins, and granzymes to directly lyse tumor cells. Cathepsin C can activate granzymes by proteolytic cleavage and granzymes can enter the target cell to induce apoptosis through various signaling pathways (125). Studies revealed Glucosamine (GlcN), an intermediate fluxing into HBP pathway to increase O-GlcNAcylation, could reduce NK cell cytotoxicity by altering the distribution of cathepsin C and E, indicating O-GlcNAcylation might play a role in regulating the cytotoxic activity of NK cells (126).

### Dendritic Cells

Dendritic cells (DCs) are a type of antigen-presenting cells which present antigens to T cells and mediate T cells activation to induce an antigen-specific immunotherapy response (127). DCs constitute a rare immune cell population within the tumor microenvironment but emerge as an essential antitumor component based on their ability to foster T cell immunity (128). Toll-like receptors (TLRs) are expressed on DCs, recognizing stimuli to activate DCs. After activation by TLRs, DCs upregulate glucose uptake with enhanced glycolytic rates (129). As HBP branches from glycolysis and forms UDP-GlcNAc to mediate protein O-GlcNAcylation, these studies point to the idea that O-GlcNAcylation might provide a favorable role in DCs activation and migration. Apart from glucose, amino acids in the environment of DCs play an important role in regulating their differentiation and activation. For example, intracellular glutamine (Gln) are enhanced in DCs after TLR receives a stimulation (127). As Gln is the substrate of HBP rate-limiting enzyme GFAT, these findings suggest O-GlcNAcylation may also involve DCs differentiation and activation.

### Neutrophils

Neutrophils are cells that carry out an innate immune response. For O-GlcNAcylation role in Neutrophils, increased O-GlcNAcylation promotes chemotaxis and cellular mobility of neutrophils (130). Studies demonstrate elevating O-GlcNAcylation levels by administration of PUGNAc, an OGA inhibitor, or GlcN activates protein Rac, an important small GTPase which plays an essential role for regulating neutrophil motility *via* activating P38 and p44/42 MAPK signaling (130).

## Concluding Remarks and Future Perspectives

Dysfunction in the O-GlcNAcylation has been associated with various chronic diseases, such as neurodegenerative diseases, diabetes and cancers (131). Thus, targeting the O-GlcNAc enzymes, OGT and OGA, or the specific O-GlcNAcylated proteins have therapeutic potential. In terms of OGA, many potent inhibitors have been developed to treat neurodegenerative disorders, such as Alzheimer’s disease (AD) since O-GlcNAcylation can compete with phosphorylation to reduce hyperphosphorylated tau aggregation in the disease background (132). Three OGA inhibitors have entered clinical trials: MK-8719 from Merck/Alectos, ASN-120290 from Asceneuron S.A., LY-3372689 from Eli Lilly, and ASN-120290 is scheduled for phase 2 clinical trial (132, 133). Due to complex expression and instability of isolated OGT, the discovery of potent OGT inhibitors is challenging. Several compounds have been developed to inhibit OGT for cancer therapy. Alloxan was the first OGT inhibitor reported but with off-target effects and general cellular toxicity (134). Pharmacological inhibition of OGT using Ac4-5SGlcNAc decreases aggressive phenotype of breast cancer cells (135). Another OGT inhibitor OSMI (OSMI-1 or OSMI-2) results in decreased tumor burden in pancreatic ductal adenocarcinoma (PDAC) and inhibit prostate cancer cells proliferation (136, 137). As both OGA and OGT are ubiquitously expressed in various tissues, therapeutic inhibition of OGT or OGA may cause global change in cellular biological process. As a result, studies aimed at obtaining specific inhibitor of key target O-GlcNAcylated proteins in a specific cancer should be an effective way for therapeutic intervention.

It is widely accepted that TME is a complex and dynamic environment, comprising of cancer cells, as well as the adjacent stromal and inflammatory cells. Due to the critical roles of the TME in regulating tumor progression, diverse cell types in TME represent targets for anticancer therapies, which is termed as TME-directed therapies, including immunotherapies (138). Cancer immunotherapy reactivates a patient’s immune system and prevent tumor immune escape. Immune checkpoint inhibitors (ICIs) are the most widely used clinically for cancer treatment. ICIs target immune checkpoint regulators to enhance anti-tumor immunity, including anti-cytotoxic T lymphocyte antigen-4 (CTLA-4), anti-programed cell death protein 1 (PD-1), and anti-PDL-1 (139). Ipilimumab (CTLA-4 inhibitor) is the first approved ICIs in 2011 and now used in the first-line setting to improve overall survival in patients with unresectable/metastatic melanoma (140). Another revolutionary immunotherapy for cancer is Chimeric antigen receptor (CAR)-T cell therapy, which genetically engineers T cells *in vitro* to possess the ability to specifically recognize and attack tumor cells (141). For anti-TAM cancer therapy, reprogramming M2 TAMs toward pro-inflammatory M1 phenotype is one strategy. Toll-like receptors (TLRs) are key players in M1 programming upon binding a ligand. Currently, Imiquimod (a TLR7 agonist) is now FDA-approved for topical administration in squamous and basal cell carcinoma (142).

However, there are still some challenges for cancer immunotherapy. For ICIs treatment, a large proportion of patients are either insensitive to ICIs or are burdened by adverse side effects. CAR-T cell therapy shows remarkable clinical responses in hematological malignancies, such as B cell leukemia or lymphoma but less efficacy in solid tumors and often cause severe toxicities, even tumor relapse (143). The therapeutic limits of immunotherapy are largely due to the network of the cells in the tumor microenvironments (TME) that operates in an immunosuppressive fashion to facilitate immune escape. Thus, an in-depth understanding of the formation of inflammatory milieu to overcome the immune suppressive TME, and finding new ways to sensitizing cancer immunotherapy are urgently needed.

Here, we focus on elucidating how O-GlcNAcylation regulates cancer-associated inflammation through identifying the role of O-GlcNAcylation in regulating precancerous inflammation lesions, inflammation-associated signaling pathways and stromal cells activation in TME. Metabolism reprogramming in TME helps sustain cancer-associated inflammation to form an inflammatory milieu to regulate cancer progression. O-GlcNAcylation acts as a metabolic sensor, linking nutrition homeostasis and cancer progression. Thus, understanding the role of O-GlcNAcylation in regulating cancer-associated inflammation and especially how site-specific O-GlcNAcylation of functional proteins in regulating inflammation-associated signaling pathways in TME is critical. Characterizing the significant roles of O-GlcNAcylation in cancer-associated inflammation may shed light on the development of new strategies targeting O-GlcNAcylated form of key proteins, thus boosting or rejuvenating immune responses against malignant cancers, and improving the therapeutic effect of cancer immunotherapy.

## Author Contributions

FD and XZ conceived and organized the manuscript. MO and CY wrote the manuscript. XD and YZ prepared the figures and contributed to the discussion. All authors have read and approved the final manuscript.

## Funding

This work was supported by grants from the National Natural Science Foundation of China (32000543), Shenzhen Science and Technology Program (Grant No. 2021A22). The funders had no role in study design, data collection and analysis, decision to publish, or preparation of the manuscript.

## Conflict of Interest

The authors declare that the research was conducted in the absence of any commercial or financial relationships that could be construed as a potential conflict of interest.

## Publisher’s Note

All claims expressed in this article are solely those of the authors and do not necessarily represent those of their affiliated organizations, or those of the publisher, the editors and the reviewers. Any product that may be evaluated in this article, or claim that may be made by its manufacturer, is not guaranteed or endorsed by the publisher.
